# Enhanced serum concentrations of transforming growth factor-beta1 in simple fatty liver: is it really benign?

**DOI:** 10.1186/1479-5876-6-72

**Published:** 2008-11-27

**Authors:** Giovanni Tarantino, Paolo Conca, Antonio Riccio, Marianna Tarantino, Matteo N Di Minno, Domenico Chianese, Fabrizio Pasanisi, Franco Contaldo, Francesco Scopacasa, Domenico Capone

**Affiliations:** 1 Federico II University Medical School of Naples, Department of Clinical and Experimental Medicine, Naples, Italy; 2 Federico II University Medical School of Naples, Department of Biomorphological and Functional Sciences, Naples, Italy; 3 Federico II University Medical School of Naples, Department of Biochemistry and Medical Biotechnology, Naples, Italy; 4 Federico II University Medical School of Naples, Department of Neurosciences, Section of Clinical Pharmacology, Naples, Italy

## Abstract

**Background:**

Inside the spectrum of non-alcoholic fatty liver disease, simple fatty liver is generally thought of as being "non progressive", differently from non-alcoholic steatohepatitis, which increases in severity due to the presence of apoptosis/inflammation and fibrosis. The "benignity" of fatty liver is widely accepted but conceptually difficult to maintain because the mechanisms underlying this entity are the same ones that determine the more severe form.

Findings provide evidence that iron overload is associated with increased liver damage and collagen deposition. Transforming growth factor-beta1 released by hepatic stellate cells during chronic liver injury plays a critical role in liver apoptosis and fibrogenesis.

**Objective:**

To verify whether both the forms of non-alcoholic fatty liver disease were really dissimilar, evaluating the serum profile of two key parameters, indexes of severity.

**Methods:**

A total of 123 patients (57 females) participated, forming three groups: forty five patients with fatty liver, 42 patients with non-alcoholic steatohepatitis and 36 with chronic hepatitis C. All had a biopsy-proven diagnosis.

**Measurements:**

Serum concentrations of transforming growth factor-beta1 and ferritin.

**Results:**

High concentrations of transforming growth factor-beta1 were noticed in patients suffering from both fatty liver and non-alcoholic steatohepatitis, 129.1 (45.4) versus 116.8 (42.2) ng/mL, P = 0.2; they were significantly superior to those of chronic hepatitis C patients 87.5 (39.5) ng/mL, P < 0.001. Ferritin levels were on average above normal values and similar in the three groups (P = 0.9), also when adjusted for gender (P = 0.5) and age (P = 0.3).

**Conclusion:**

No difference between serum concentrations of transforming growth factor-beta1 and ferritin in fatty liver and non-alcoholic steatohepatitis suggests that these forms share more common aspects, regarding their progression, than previously thought.

## Background

Non-alcoholic fatty liver disease (NAFLD) represents a complex of liver diseases that range from simple fatty liver (FL), at the most clinically benign end of the spectrum, through an intermediate, generally progressive lesion, non-alcoholic steatohepatitis (NASH) to cirrhosis, at the opposite end. Diagnosis of NAFLD can usually be done by imaging studies in absence of other liver disease. Liver biopsy is required to size disease severity (inflammation, degenerative lesion and fibrosis), even though some limitations cast doubts on its use in clinical settings [1].

The definition of "benignity" concerning FL is wide-accepted [2] but conceptually difficult to maintain because the mechanisms, i.e., insulin resistance (IR), underlying this entity are the same ones that determine the more severe form.

The key process in the progression of NAFLD from the very beginning to the end is fibrosis. An animal model of "fibrosing steatohepatitis" that replicates the histologic features of human NASH stresses the sequence of steatosis, inflammatory cell injury and fibrogenesis, mediated by hepatic stellate cells (HSCs) via up-regulation of transforming growth factor-beta1 (TGF-β1) [3]. An alternative pattern is followed by leptin that facilitates proliferation and prevents apoptosis of HSCs [4].

There is an increasing body of evidence that iron overload is associated with metabolic syndrome (MS) and NAFLD [5]. Observation of liver fibrosis in a rat model of NASH suggests that iron induces increase in hepatocytes apoptosis and contributes to the development of fibrosis directly or indirectly via induction of TGF-β1 production in hepatocytes and macrophages at an earlier time than expected [6]. It is important to stress that hepatocyte apoptosis is significantly increased in patients with NASH and correlates with disease severity [7].

TGF-β1 is a profibrotic cytokine whose action is mediated by Smad proteins and p38 MAPK. They have been found to independently and additively regulate α1(I) collagen gene expression by transcriptional activation, while p38 MAPK, but not Smad signaling, increases α1(I) collagen mRNA stability leading to increased synthesis and deposition of type I collagen [8].

As previously reported, histology, which has not probably an optimal sensitivity and specificity, leads to biased accuracy estimates and gives a frozen-in-time picture. An approach to look into the supposed "benignity" of FL and "progressivity" of NASH is to speculate about eventual differences/similarities in mechanisms between the two entities. With this in mind, we tracked in a NAFLD cohort the behaviour of serum TGF-β1, an indirect severity progression index, and ferritin, an ancillary marker for IR, correlating their concentrations to those present in chronic hepatitis C (CHC), disease characterized by the combination of apoptosis/inflammation and fibrosis, in which TGF-β1 and iron overload could play a key role too [9,10].

## Methods

### Population

**Figure 1:**
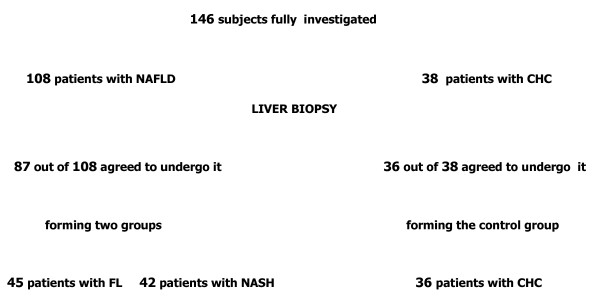
**The flow of participants through the study**. NAFLD, non-alcoholic fatty liver disease; FL, fatty liver; NASH, non-alcoholic steatohepatitis; CHC, chronic hepatitis C.

One hundred and forty six adult Caucasian patients from the beginning of 2005 to the end of 2007 were consecutively investigated at our Department (Figure 1) in a cross-sectional fashion.

Every patient gave his or her informed consent to this study, which had been approved by the local Ethics Committee.

### NAFLD patients

We enrolled 108 patients who fulfilled the following inclusion criteria: presence of overweight/obesity and visceral adiposity, associated with recent US features of "bright liver", with or without aminotransferases increase of unknown origin.

Subjects were classified as being overweight or as having first degree obesity on the basis of body mass index (BMI) cut-off points of ≥ 25.0 and ≤ 29.9, or > 29.9 and ≥ 34.9 kg/m^2^, respectively. Central obesity was identified by waist circumference (WC) > 102 cm in men or > 88 cm in women, measured at the midpoint between the lower border of the rib cage and the iliac crest. Metabolic syndrome (MS) was defined according to the revised Adults Treatment Panel III (2001), and three or more criteria were considered: plasma glucose concentration of at least 100 mg dL^-1^, WC > 102 cm in men and > 88 cm in women, serum high-density lipoprotein (HDL)-cholesterol concentration < 40 mg dL^-1 ^in men and < 50 mg dL^-1 ^in women, blood pressure of at least 130/85 mm Hg, and serum triglyceride concentration of at least 150 mg dL^-1^.

IR was calculated by modified homeostasis model assessment-index (HOMA), with the following formula: fasting insulin (μU/mL) * plasma glucose (mg/dL)/405 [11].

Exclusion criteria were a recent history of acute inflammation (very high, ≥ 4 times the upper limit of normality, values of C reactive protein, CRP); presence of hepatitis B and C, neoplastic and/or haematological diseases, autoimmune and storage diseases; prior (at least 3 months) use of drugs inducing hepatic steatosis or affecting inflammation or angiotensin-converting enzyme inhibitors/angiotensin II type 1 receptor blockers. Alcohol abuse was ruled out according to the DSM-IV diagnostic criteria, by means of screening tests such as MAST (Michigan Alcohol Screening Test) and CAGE (Cut down, Annoyed, Guilty, and Eye opener) [12], as well as random tests for blood alcohol concentration and the use of a surrogate marker, e.g., mean corpuscular volume. Patients on antihypertensive therapy maintained a balanced medical regimen throughout the study.

Eighty-seven out of 108 patients initially selected agreed to perform liver biopsy. On the basis of the results of hepatic histology, 42 patients (19 females) were assigned to the NASH group and 45 (21 females) to the FL one. Steatohepatitis was graded on the basis of the degree of macrovesicular steatosis, mixed lobular inflammation and hepatocyte ballooning, using a composite NAFLD activity score (NASH, > 5) [13]. The presence of perisinusoidal fibrosis was noted and scored as none, rare, mild or moderate. Early fibrosis was distinguished from advanced fibrosis based on the presence of bridging fibrosis (score of 3 or more).

### Chronic hepatitis C patients

Thirty-eight individuals were diagnosed as to have elevated values of serum alanine aminotransferase (ALT) for at least six months. These subjects possessed detectable serum HCV-RNA (COBAS AmpliScreen HCV Test, v2.0, with automated amplification and detection using polymerase chain reaction method on the COBAS AMPLICOR Analyzer, Roche; the lower detection limit was 200 IU/mL), before starting antiviral treatment. Thirty-six patients (17 females) underwent liver biopsy. Histological features were evaluated using the Ishak scoring system for inflammation and fibrosis [14]. In brief, inflammation was scored using four parameters (periportal or periseptal interface hepatitis, confluent necrosis, focal lytic necrosis and portal inflammation) to obtain a histological activity index (HAI, maximum score 18), and fibrosis was scored as 0 – 6. The selected patients' tissue specimens were considered adequate for evaluation when at least four portal (or septal) areas were available for review and if they had length superior to 1.5 cm.

### Ultra Sonography

Determinations were made by two expert operators, blinded to each other, using an ultrasound (US) diagnostic system (ESAOTE, Genoa, Italy) with a 3.5-MHz convex probe. The classification of "bright liver" was based on the following scale of hyperechogenity: 0 = absent, 1 = light, 2 = moderate, 3 = severe.

### Analytes

CRP was dosed by an enzyme immunoassay kit of BioCheck, Inc, Foster City, CA, USA.

TGF-β1 was dosed by using Quantikine immunoassay kit from R&D Systems, Inc. Minneapolis, MN, USA. Serum separator tubes were used to allow samples to clot for 30 minutes at room temperature. For complete release of TGF-β1, samples were incubated overnight at 2 – 8°C before centrifugation for 15 minutes at 1000 × g. Removed serum was stored at -70°C.

The intra-assay and inter-assay precision coefficient variation was 3.4% and 8.4%, respectively. TGF-β1 levels in 15 controls showed a median of 26.9 ng/mL, and 5^th ^– 95^th ^percentile 23.0 – 34.0. The mean minimum detectable was 4.87 pg/mL.

For the determination of ferritin in serum was used the Ferritin ELISA Quantitation kit by GenWay Biotech, Inc. San Diego, CA 92121. The minimum detectable ferritin concentration by the assay was 5.0 ng/mL. Normal values were in males 20–370 ng/mL and in females 10–150 ng/mL. The inter-assay coefficients of variation were 4.2%, 5.1% and 6.6% at the concentrations of 37, 221 and 340 ng/mL, respectively, whereas the intra-assay coefficients of variation were 3.5%, 5.7% and 3.6% at the same concentrations, respectively.

Data collection of sonographic parameters was done before the histological classification, whereas serum ferritin and growth factor concentrations were obtained on stored samples.

The liver biopsy, blood samples and US parameters were strictly carried out within two months in order to lessen potentially confounding lifestyle changes or intercurrent illnesses.

### Statistics

Variables normally distributed (Kolmogorov-Smirnov test) such as age (P = 0. 13), ferritin (P = 0.12), ALT (P = 0.1), CRP (P = 0.07) and TGF-β1 (P = 0.054) were expressed as mean (SD). BMI, not normally distributed (P = 0.003), and ordinals, i.e., US and histology scores, were expressed as median and range.

The t test or ANOVA and the Mann-Withey test or Kruskal-Wallis were adopted to compare means or median, respectively. The pairwise analysis of subgroups, post-hoc comparisons after ANOVA, was obtained by the Tukey test. Furthermore, the ANCOVA was used to control for factors. The chi square was performed to look for differences in the classification system. Tracking the degree of association between single parameters in each group, Pearson's r or Spearman's rho was chosen according to the variable distribution (normal or not normal as well as being ordinals, respectively). Statistical analysis was performed operating on Systat 12 and MedCalc Version 9.4. software packages.

## Results

The biopsy-proven selected population (Table 1) was well balanced for gender (Chi-square = .03, P = 1), and obviously not for BMI (Kruskal-Wallis, P = < 0.001). MS was indifferently represented across the NAFLD groups (20 out of 45 in FL and 25 out of 42 in NASH, Chi-square = 1.3, P = 0.2).

**Table 1:** Main laboratory data and characteristics of the studied population

	Diagnosis								
	**CHC n 36**			**FL n 45**			**NASH n 42**		
	Gender	Mean	SD	Gender	Mean	SD	Gender	Mean	SD
**Age**		44.5	10.0		42.3	9.1		40.4	10.5
**TGF-β1 **ng/mL*		87.5	39.5		129.1	45.4		116.8	42.2
**CRP**					0.8	0.4		0.9	0.4
**ALT **U/L^#^		72.7	25.6		49.2	17.8		54.3	21.8
**Ferritin **ng/mL	17 F	203.6	86.5	21 F	232.3	117.4	19 F	279.8	150.3
**Ferritin **ng/mL	19 M	396.4	153.5	24 M	381.5	137.2	23 M	357.2	132.3
**Waist Circumference **(cm)				21 F	98	6.1	19 F	100	4.7
**Waist Circumference **(cm)				24 M	105	6.3	23 M	108	7.7
**HOMA**					3.2	1.4		3.4	1.5
		Median	Range		Median	Range		Median	Range
**BMI**‡		26	22–30		29	27–32		29	27–32
**Fibrosis score**		1	1–3					1	1–2
**US steatosis Score**		2	1–3					1	1–3

CHC, chronic hepatitis C; FL, fatty liver; NASH, non-alcoholic steatohepatitis; ALT, alanine aminotransferase; CRP, C reactive protein; TGF-β1, transforming growth factor beta1; BMI, body mass index; HOMA, homeostasis model assessment-insulin resistance index.

* P < 0.001, FL versus CHC and 0.008, NASH versus CHC patients; ^# ^P < 0.001, CHC versus FL and NASH patients; ‡ P < 0.001, CHC versus FL and NASH patients.

In NAFLD patients we found high TGF-β1 concentrations. No statistically significant difference was found between FL and NASH subgroups (P = 0.2).

TGF-β1 levels were more increased in FL and in NASH patients than CHC patients; this difference disappeared when data were adjusted for age.

Ferritin levels were found elevated and not different in the three groups (P = 0.9), also when adjusted for gender (P = 0.5) and age (P = 0.3).

**Figure 2:**
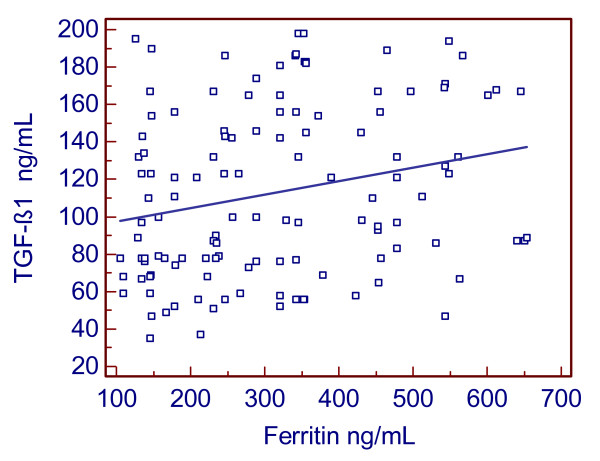
**Association between serum TGF-β1 and ferritin**. TGF-β1, transforming growth factor-beta1.

Serum TGF-β1 was significantly correlated to serum ferritin considering the full population (r = 0.23, P = 0.009, Figure 2), especially in female patients (r = 0.5, P < 0.001), in NASH female patients (r = 0.45, P = 0.048) and in FL patients of both gender (r = 0.35, P = 0.02), whereas in all the CHC patients there was only a certain trend (r = 0.32, P = 0.05). No association was found between CRP and TGF-β1 (P = 0.8). ALT activity showed a negative association with TGF-β1 levels (r = -0.34, P = 0.026) in NASH patients and none in CHC patients (P = 0.9). Further details are shown in Table 2 and 3.

**Table 2:** Correlations between ferritin and other parameters

	**CHC**	**FL**	**NASH**
TGF-β1	Ferritin (17 females, F)	Ferritin (21 F)	Ferritin (19 F)
	r = 0.35	r = 0.5	r = 0.46
	P = 0.16	P < 0.001	P = 0.048

TGF-β1	Ferritin (19 males, M)	Ferritin (24 M)	Ferritin (23 M)
	r = 0.06	r = 0.08	r = -0.26
	P = 0.78	P = 0.8	P = 0.23

CRP	ND	Ferritin (F)	Ferritin (F)
		r = 0.13	r = 0.36
		P = 0.57	P = 0.13

CRP	ND	Ferritin (M)	Ferritin (M)
		r = -0.49	r = -0.08
		P = 0.01	P = 0.7

ALT	Ferritin (females)	Ferritin (F)	Ferritin (F)
	r = 0.36	r = 0.04	r = -0.17
	P = 0.18	P = 0.85	P = 0.48

ALT	Ferritin (M)	Ferritin (M)	Ferritin (M)
	r = 0.42	r = -0.01	r = 0.42
	P = 0.07	P = 0.95	P = 0.045

BMI	Ferritin (F)	Ferritin (F)	Ferritin (F)
	rho = -0.13	rho = -0.05	rho = 0.14
	P = 0.59	P = 0.8	P = 0.5

BMI	Ferritin (M)	Ferritin (M)	Ferritin (M)
	rho = -0.24	rho = 0.3	rho = -0.22
	P = 0.3	P = 0.14	P = 0.3

US score	ND	Ferritin (F)	Ferritin (F)
		rho = -0.19	rho = -0.24
		P = 0.4	P = 0.31

US score	ND	Ferritin (M)	Ferritin (M)
		rho = -0.04	rho = -0.24
		P = 0.85	P = 0.3

Fibrosis score	Ferritin (F)	ND	Ferritin (F)
	rho = 0.14		rho = -0.51
	P = 0.37		P = 0.03

Fibrosis score	Ferritin (M)	ND	Ferritin (M)
	rho = 0.18		rho = 0.20
	P = 0.44		P = 0.34

Age	Ferritin (F)	Ferritin (F)	Ferritin (F)
	r = -077	r = -0.34	r = -0.01
	P = 0.0003	P = 0.13	P = 0.68

Age	Ferritin (M)	Ferritin (M)	Ferritin (M)
	r = 0.40	r = 0.29	r = -0.26
	P = 0.08	P = 0.16	P = 0.23

CHC, chronic hepatitis C; FL, fatty liver; NASH, non-alcoholic steatohepatitis; ALT, alanine aminotransferase; CRP, C reactive protein; TGF-β1, transforming growth factor beta1; BMI, body mass index; US score, hepatic steatosis score at ultrasound; r, Pearson's correlation coefficient and rho, Spearman's rank correlation coefficient, chosen according to the variable distribution (normal or not normal as well as being ordinals, respectively); ND, not determined; F, females; M, males.

**Table 3:** Correlations between TGF-β1 and other parameters

	**CHC N = 36**	**FL N = 45**	**NASH N = 42**
CRP	ND	TGF-β1	TGF-β1
		**r **= 0.37	r = 0.29
		P = 0.08	P = 0.055

ALT	TGF-β1	TGF-β1	TGF-β1
	r = -0.02	r = -0.13	r = -0.34
	P = 0.9	P = 0.38	P = 0.026

BMI	TGF-β1	TGF-β1	TGF-β1
	rho = 0.13	rho = 0.18	rho = 0.09
	P = 0.46	P = 0.22	P = 0.56

US score	ND	TGF-β1	TGF-β1
		rho = -0.08	rho = 0.05
		P = 0.57	P = 0.72

Fibrosis score	TGF-β1	ND	TGF-β1
	rho = -0.09		rho = 0.05
	P = 0.60		P = 0.75

Age	TGF-β1	TGF-β1	TGF-β1
	r = -0.02	r = 0.02	r = -0.009
	P = 0.9	P = 0.9	P = 0.9

CHC, chronic hepatitis C; FL, fatty liver; NASH, non-alcoholic steatohepatitis; ALT, alanine aminotransferase; CRP, C reactive protein; TGF-β1, transforming growth factor beta1; BMI, body mass index; US score, hepatic steatosis score at ultrasound; r, Pearson's correlation coefficient and rho, Spearman's rank correlation coefficient, chosen according to the variable distribution (normal or not normal as well as being ordinals, respectively); ND, not determined.

An inverse relationship was present between fibrosis score and serum TGF-β1 (rho = -0.27, P < 0.001).

High HOMA values were associated with high serum TGF-β1 levels (r = 0.48, P < 0.001).

US steatosis score well correlated to WC in women (rho = 0.58, P < 0.001) as well as in men (rho = 0.61, P < 0.001).

## Discussion

The key findings we provide are briefly i) subjects with FL and NASH exhibit quite the same elevated values of serum TGF-β1, both greater than those present in CHC patients; ii) there is a fair correlation between levels of this cytokine and ferritin in FL patients.

Our data somehow disagree with the body of present knowledge. In fact, they provide evidence for the idea that, being fibrosis the key process that distinguishes the non-progressive from the progressive form of NAFLD and having found a marker of fibrosis well represented in FL patients, FL should not be considered a benign disease yet. Further, we failed to confirm the crucial role of CRP in differentiating FL from NASH even though NASH patients revealed the highest concentrations [15].

Discussing possible mechanisms and explanations for our findings, we emphasize that TGF-β1-induced fibrosis in organ pathology and dysfunction appears to be increasingly relevant to a variety of distinct diseases [16].

Enhanced serum TGF-β1 concentrations could represent a marker of early activation of mesenchymal HSCs. This interpretation is strengthened by the findings of a negative correlation of serum TGF-β1 with fibrosis score, feature of stable collagen deposition, and by a good correlation between the same cytokine and serum ferritin. In fact, liver iron deposits in CHC are common and associated with activation of HSCs, ultimately contributing to liver damage [10,17].

Increasing evidence suggests hepatocyte apoptosis, due to increased oxidative stress, is a key mediator of liver injury in NAFLD [18]. But, is apoptosis restricted to hepatocytes alone? It is likely that, in an initial phase, apoptosis also acts on activated HSCs decreasing the collagen fibres [19]. This could happen in FL. Successively, this mechanism does not prevent the waterfall effect of hepatic fibrosis, characteristic feature of NASH. Alternatively, being the deposition and degradation of hepatic fibrous tissue a dynamic equilibrium course, increased expression patterns of matrix-metalloproteinases -1, -2, -3, and tissue inhibitors of metalloproteinases -1 and -2 genes could promotes the degradation of extra-cellular matrix in an early step, such as in FL. Anyway, the mechanisms remain to be further studied.

Although our results are referred to a larger population, we are not able to confirm that high levels of plasma TGF-β1 represent a possible method of diagnosing NASH in NAFLD patients [20].

We found that the criterion of liver enzymes increase, widely used to separate NASH from FL, is vanishing according to a recent study in which 25 out of 64 (39%) patients with biopsy-proven FL was found to have ALT levels superior to 30 U/L [21]. In addition, having found no or negative correlation between ALT activity and TGF-β1 levels in NASH and CHC patients, respectively, suggests that TGF-β1 is related to apoptosis rather than to inflammation.

Still, discussing other limitations, we should ask some questions.

Firstly, does a randomized determination mirror the "at steady state" serum concentration of this cytokine?

TGF-β1 differs from the majority of growth regulatory factors since it is generally synthesized and secreted in a biologically latent form, and this must be activated before TGF-β1 can exert its biological effects on target cells. TGF-β1 in this latent complex had a long plasma half-life (more than 100 min). Having found elevated values of serum TGF-β1 in FL, it is likely that a hepatic over-expression of the same cytokine is present. The only one serum determination for each patient is a "snapshot in time" methodology, but this is understandable; this alone with small numbers of patients in the 3 subgroups limits any definitive conclusion that can be drawn from this study.

Secondly, why are not evident histological features of fibrosis in FL patients undergone liver biopsy?

Our findings do not represent an isolate case. In fact, HSC activation was not correlated to HAI and fibrosis score, valued by Knodell and Batts separate systems, in a subset of patients who developed severe hepatitis C recurrence on 4-month after liver transplantation [22]. The interpretation could be that we similarly faced an early fibrogenesis that would have been apparent across a long period of time, making the TGF-β1, indirect marker of HSC activation, better useful in predicting the subsequent hepatic fibrosis.

We could have carried out other markers of fibrosis for the noninvasively staging of NAFLD patients [23]. However, to date, none of these markers have been independently validated in different populations in a prospective way. Moreover, all of these studies have been tested in a cross-sectional fashion, and then the role of these biomarkers for monitoring disease progression remains completely unknown.

To let us get down to the grassroots of the FL benignity from the beginning we should relay on longitudinal observations lasting several years, but this approach is difficult to plan. Crucial future research directions could be assessing liver fibrosis in FL patients during a long period of time by using non invasive tools such as fibrosis markers or elastograghy.

## Conclusion

Similar levels of TFβ1 and ferritin in fatty liver and non-alcoholic steatohepatitis suggest that these forms share some common aspects, regarding their progression. If FL can evolve in liver cirrhosis, factors underlying this illness should be more intensively corrected, representing NAFLD also an epiphenomenon of MS, which has become a major health problem.

## Abbreviations

(NAFLD): Non-alcoholic fatty liver disease; (FL): fatty liver; (NASH): non-alcoholic steatohepatitis; (IR): insulin resistance; (CHC): chronic hepatitis C; (TGF-β1): transforming growth factor-beta1; (HSCs): hepatic stellate cells; (MS): metabolic syndrome; (BMI): body mass index; (CRP): C reactive protein; (WC): waist circumference; (HOMA): homeostasis model assessment-index; (MAST): Michigan alcohol screening test; (CAGE): cut down, annoyed, guilty, eye opener; (ALT): alanine aminotransferase.

## Competing interests

The authors declare that they have no competing interests.

## Authors' contributions

FS and DCa carried out the laboratory analyses. MT performed the ultrasound studies. DCh, MND, PC, FP, AR and FC participated in the design of the study and in drafting the manuscript. GT conceived of the research, was the main investigator and the coordinator of the study, made the statistics and drafted the manuscript. All authors read and approved the final manuscript.
